# Radiological features of Stafne mandibular bone cavity in panoramic image and cone beam computed tomography

**DOI:** 10.1186/s40902-024-00415-y

**Published:** 2024-03-19

**Authors:** Jangwoo Son, Dong-Jae Lee, Kang-Min Ahn

**Affiliations:** Department of Oral and Maxillofacial Surgery, College of Medicine, Asan Medical Center, University of Ulsan, Seoul, South Korea

**Keywords:** Stafne mandibular bone cavity, Mandible, Cone-beam computed tomography, Salivary gland

## Abstract

**Background:**

Stafne mandibular bone cavity (SMBC) is an asymptomatic radiolucent lesion observed in the mandible on X-ray images, with well-defined borders. This lesion does not require treatment, and size changes are very rare. The purpose of this article is to summarize the radiological and clinical features of SMBC, aiming to prevent misinterpretation of this benign lesion as other pathologies and aid in differential diagnosis within the field of dental practice.

**Methods:**

A total of 32 patients who visited our hospital and were radiologically diagnosed with SMBC based on cone-beam computed tomography (CBCT) and panoramic images between 2005 and 2021 were included in this study. Among them, surgical intervention was performed in one case. Gender and age distribution of the patients, radiographic location and size of the lesion, relationship with the mandibular canal in CBCT, presence of cortical bone erosion in the mandibular lingual area in panoramic images, and the amount of residual cortical bone on the affected side in CBCT were investigated.

**Result:**

Patients were 26 men (81.3%) and 6 women, with a mean age of 54.3 years. The average horizontal and vertical length was 16.6 mm and 10.6 mm. Out of a total of 32 cases, 29 cases were found in the posterior region of the mandibular body, while 3 cases were in the angle of the mandible. Lesions located below the mandibular canal were observed in 29 cases, while lesions involving the mandibular canal were present in 3 cases. Erosion of the mandibular lingual cortical bone was observed in 11 cases (34.4%), while 21 cases (65.6%) showed no erosion on panoramic images. Among the total of 14 cases (43.8%) where the cortical bone on the affected side was invaded, the average residual cortical bone thickness was 1.1 mm.

**Conclusion:**

SMBC is a benign lesion primarily found in the mandibular angle and posterior body of the mandible. In most cases, treatment is not necessary, and differentiation from other lesions can be achieved by understanding its clinical characteristics and features on panoramic radiographs and CBCT.

## Background

Stafne described a series of asymptomatic radiolucent lesions located near the angle of the mandible in 1942 [1]. Similar lesions were reported successively, round or oval concavity of the lingual surface of the mandible, and these were named “Stafne bone cyst,” “Lingual mandibular salivary gland depression,” “Latent bone cyst,” “Static bone defect,” and “Lingual cortical mandibular defect.” The lesion, bearing various names, differs from a true cyst in that it lacks an epithelial lining [1, 2]. In the pseudocysts or osseous cavities, various components, including salivary gland tissue, muscles, lymphatic tissue, blood vessels, adipose tissue, and connective tissue, can be identified [2]. Hence, to avoid confusion with a true cyst, the nomenclature for this lesion has been designated as “Stafne mandibular bone cavity (SMBC)” in the International Classification of Diseases 11th Edition.

SMBC is primarily found in individuals in their 40 s and 50 s, with a higher prevalence in males compared to females. The main sites of occurrence are the mandibular angle or the posterior region of the mandible, inferior to the mandibular canal. On panoramic radiographic images, SMBC typically appears as a radiolucent lesion with a round to ovoid shape, exhibiting radiolucency with a sclerotic border, resembling a cyst [3]. In cone-beam computed tomography (CBCT) images, SMBC is observed as an oval-shaped space filled with connective tissue, accompanied by lingual cortical bone perforation, typically located in the posterior region of the mandible.

The etiology of SMBC is not yet fully understood, but there are two main hypotheses. One suggests that it occurs congenitally when the mandible is forming, supporting SMBC's static nature. This hypothesis explains that it may occur due to the congenital entrapment of the inferior salivary gland into the mandible or insufficient bone resorption in the space previously occupied by Meckel’s cartilage [4]. The other hypothesis suggests that SMBC may develop through a gradual process, where the pressure from the sublingual gland, pulsation of the facial artery, and other factors lead to bone resorption on the lingual aspect of the mandible, resulting in the formation of SMBC [4, 5]. However, regardless of the etiology, both hypotheses support that SMBC is a benign lesion that does not require treatment, emphasizing the need to distinguish SMBC from other conditions requiring intervention.

Dentists typically learn how to distinguish these pseudocysts from radiologically similar pathology during their undergraduate dental education. However, in actual clinical practice, many dentists frequently misdiagnose SMBC as other lesions due to radiological similarities [6]. Such misdiagnoses can lead to unnecessary additional examination and increased costs for patients, and in some cases, even unnecessary surgical intervention, resulting in iatrogenic complications. To avoid these situations, it is essential to have a good understanding of the radiological, anatomical, and clinical characteristics of SMBC.

Therefore, the objective of this study is to compile the radiological and clinical characteristics of 32 cases of SMBC diagnosed in a single hospital to aid general dentists in distinguishing SMBC from other conditions.

## Methods

A total of 32 patients who visited a single hospital and were radiologically diagnosed with Stafne bone cysts based on CBCT and panoramic images between 2005 and 2021 were included in this study. Among them, surgical intervention was performed in one case. Gender and age distribution of the patients, radiographic location and size of the lesion, relationship with the mandibular canal in CBCT, presence of cortical bone erosion in the inferior border of the mandible in panoramic images, and the amount of residual buccal cortical bone on the affected side in CBCT were investigated. The amount of residual buccal cortical bone was classified according to the newly established classification method (Table 1). In the case of increased size with age, surgery was conducted. Patient ages were determined based on the age at the initial detection of the lesion. Lesion size was defined as the horizontal length of the lesion parallel to the mandibular body, and the vertical length perpendicular to this horizontal length. Histopathological results of the one case undergoing surgery were obtained from the department of pathology (IRB No. 2023–1251).

**Table 1:** New classification of the amount of buccal cortical bone on the affected side in CBCT

Type	Amount of buccal cortical bone on the affected side in CBCT
Type I	Thickness of buccal cortical bone > 1.3 mm
Type II	0 mm < thickness of buccal cortical bone ≤ 1.3 mm
Type III	Thickness of buccal cortical bone = 0 mm (perforation)

## Result

Among a total of 32 participants, 26 were male, resulting in a higher occurrence rate among males (81.25%) compared to females. The average age at the initial detection of Stafne bone cysts was 54.3 years. The average age for males was 54.5 years, and for females, it was 53.3 years. The age distribution consisted of 1 case in the 20 s, 2 cases in the 30 s, 8 cases in the 40 s, 9 cases in the 50 s, 9 cases in the 60 s, and 3 cases in the 70 s, with the most common occurrence in the 50 s to 60 s age group, respectively. The minimum age was 26 years, and the maximum age was 71 years.

**Fig. 1:**
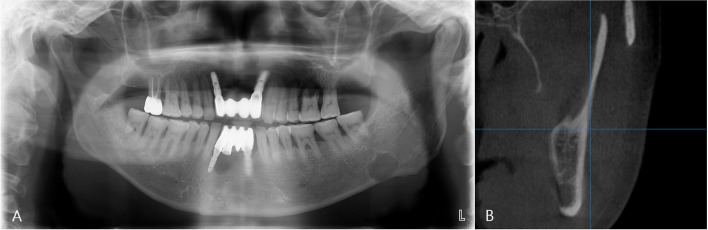
Type I SMBC (**A**) panoramic radiograph showing SMBC on the left mandibular body. **B** The CBCT scan demonstrates that SMBC does not invade the buccal cortical bone on the left side of the mandible and remains intact over a thickness of 1.3 mm

**Fig. 2:**
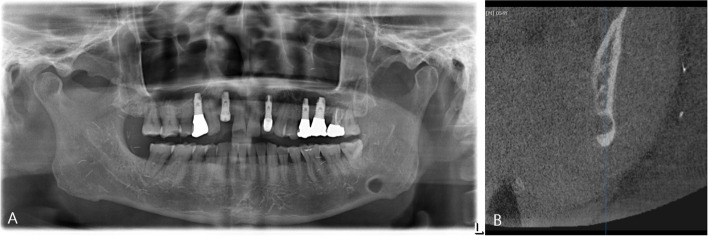
Type II SMBC (**A**) panoramic radiograph showing SMBC on the left mandibular body. **B** The CBCT scan demonstrates that SMBC invaded the buccal cortical bone on the left side of the mandible, but it has not yet perforated

**Fig. 3:**
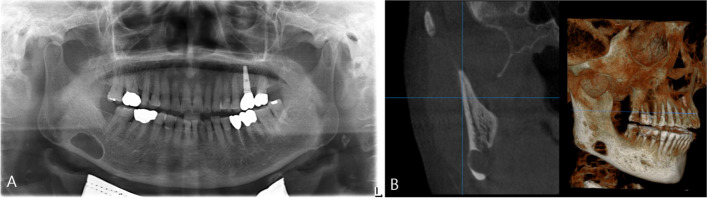
Type III SMBC (**A**) panoramic radiograph showing SMBC on the right mandibular body. **B** The CBCT scan demonstrates the expansion and perforation of the buccal cortical bone on the right side of the mandible due to the involvement of SMBC

**Fig. 4:**
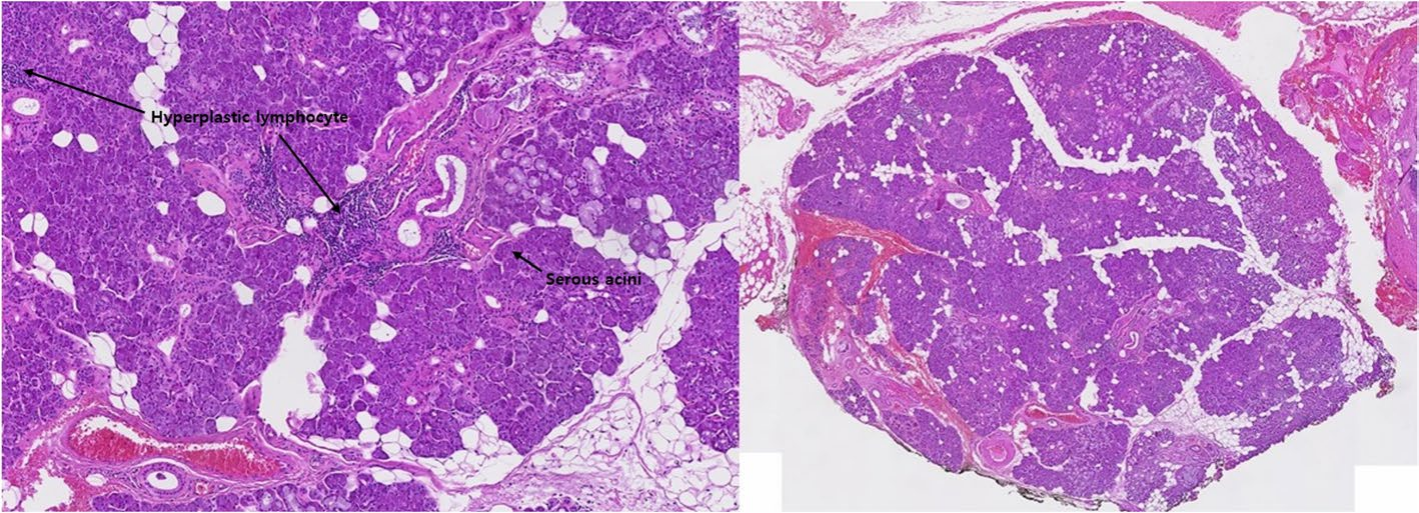
In a single case where surgery was performed, the excisional biopsy identified lymphocytic sialadenitis, including reactive hyperplastic lymph nodes. Salivary duct and serous acini are observed in the histological images

The average horizontal length was 16.6 mm, and the average vertical length was 10.6 mm. The largest lengths recorded in the horizontal and vertical lengths were 32.4 mm and 17.5 mm, respectively, in a single case. Among the cases where the lesion was found on the left side, 18 were observed, with 16 located in the body of the mandible, inferior to the second molar, and 2 in the angle of the mandible. On the right side, there were a total of 14 cases, with 13 located in the body of the mandible and 1 in the angle of the mandible. Lesions located below the mandibular canal were observed in 29 cases, while lesions involving the mandibular canal were present in 3 cases. Erosion of the inferior border of the mandible was observed in 11 cases (34.4%), while 21 cases (65.6%) showed no erosion on panoramic images. There was a total of 14 cases (43.8%) where the cortical bone on the affected side was invaded, and among these, there were 4 cases with cortical bone thickness of less than 0.5 mm. The average residual cortical bone thickness after cortical bone invasion on the affected side was 1.10 mm. Upon applying the newly established classification method in this study, the results revealed 18 cases classified as type I, 12 cases as type II, and 2 cases as type III. Representative cases for each type were provided as examples (Figs. 1, 2, and 3). The excisional biopsy revealed findings of lymphocytic sialadenitis, including reactive hyperplastic lymph nodes (Fig. 4). The overall results are summarized in Table 2.

**Table 2:** Summary of patients with demographic data, radiographic characteristics, and classification

Variable			
Age	Average (age)		54.3
	Age range (age)		26–71
Gender	Male (No)		26
	Female (No)		6
Size	Average horizontal length (mm)		16.6
	Average vertical length (mm)		10.57
Location	Left side	Body of mandible (No)	16
		Angle of mandible (No)	2
		Total (No)	18
	Right side	Body of mandible (No)	13
		Angle of mandible (No)	1
		Total (No)	14
Relationship with mandibular canal	Below of the mandibular canal (No)		29
	Involving the mandibular canal (No)		3
Presence of cortical bone erosion of the inferior border of the mandible	No (No)		11
	Yes (No)		21
Amount of buccal cortical bone	Type I (No)		18
	Type II (No)		12
	Type III (No)		2

*No* number

## Discussion

SMBC is rare, with reported incidence rates ranging from 0.1 to 0.48% in the literature [7, 8]. However, its consistent detection has been increased recently due to the increased utilization of dental services and the widespread use of panoramic radiography. In most SMBC cases, patients are asymptomatic, and the lesions are typically identified in panoramic radiographs by chance. In this study, lesions were discovered in panoramic radiographs taken for other reasons in all cases except for one instance where surgery was performed. Notably, all patients in the study had any discomfort or symptoms. Previous literature indicates that the incidence rate of SMBC is reported to be 4.0 to 5.0 times higher in males compared to females, based on gender differences [1, 9, 10]. In the study conducted by Philipson et al. [11], SMBC was found to have a higher incidence in males at a ratio of 6:1, while Morita et al. [12] reported that SMBC predominantly occurs in males at a ratio of 7:3. This gender difference corresponds to the results of our study, demonstrating a male predominance with a prevalence of 81.25%, compared to 18.75% in females, in line with a higher incidence of SMBC in males. SMBC is rarely observed in individuals between the ages of 10 s and 30 s, and it is predominantly detected in individuals aged 40 s and above, particularly in those in their middle-aged and older years [3, 11]. In this study, the average age at which SMBC was detected was 54.3 years, consistent with the previous research that SMBC is predominantly found in middle-aged and older populations. Notably, cases of SMBC in individuals under 30 s were rare, accounting for only 3 cases (9.38%), and no instances were observed in 10 s. This pattern, where SMBC is rarely identified during childhood and is predominantly detected in middle-aged and older individuals, provides support for the hypothesis that SMBC develops as a consequence of a progressive process rather than being congenital in nature.

**Fig. 5:**
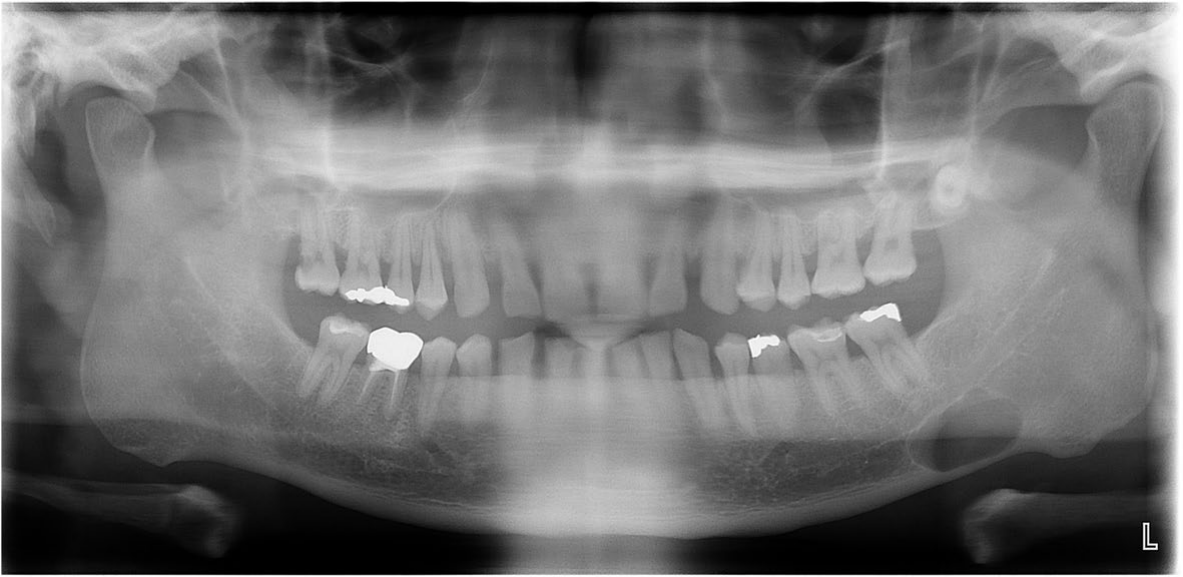
The continuity of the mandibular border was maintained even when the horizontal length exceeded 30 mm

**Fig. 6:**
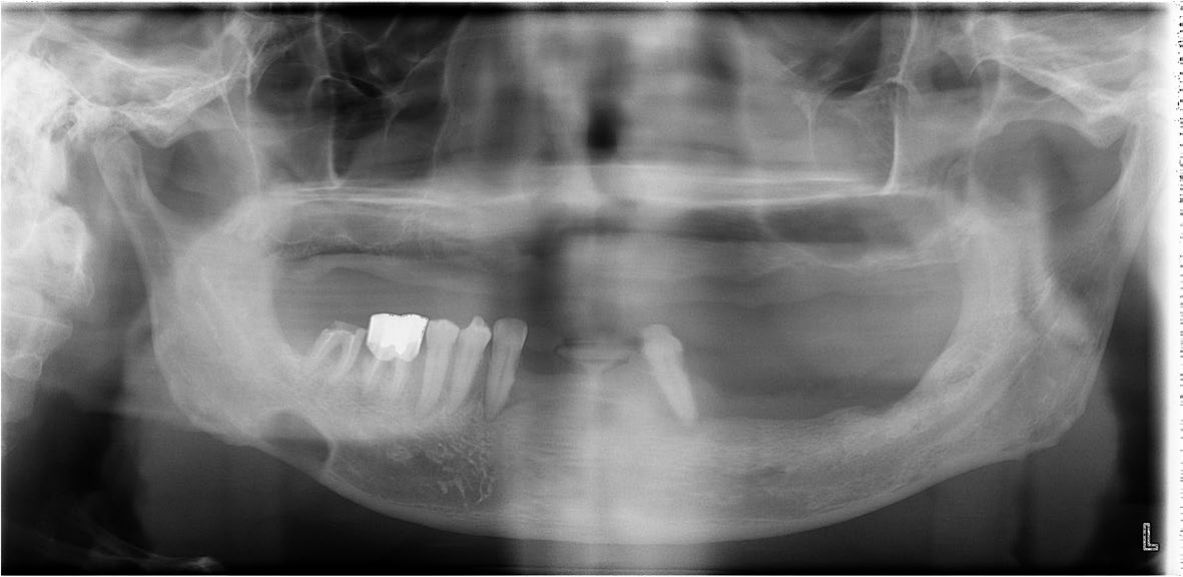
The continuity of the mandibular border was disrupted even when the horizontal length was less than 30 mm

Previous studies have reported that the size of the bone defect in SMBC is typically in the range of 1–3 cm in diameter on panoramic images. It has been noted that when the horizontal length exceeds 3 cm, the continuity of the mandibular border may be interfered with, allowing palpation of the lesion from the overlying skin [13, 14]. In this study, the size of SMBC was measured, with an average horizontal length of 16.6 mm, an average vertical length of 10.6 mm, and a maximum diameter of 32.4 mm. These measurements are in accordance with previous research findings. However, there were cases in which the continuity of the mandibular border was maintained even when the horizontal length exceeded 30 mm (Fig. 5). Conversely, instances were also observed where the continuity of the mandibular border was disrupted even when the horizontal length was less than 30 mm (Fig. 6).

**Fig. 7:**
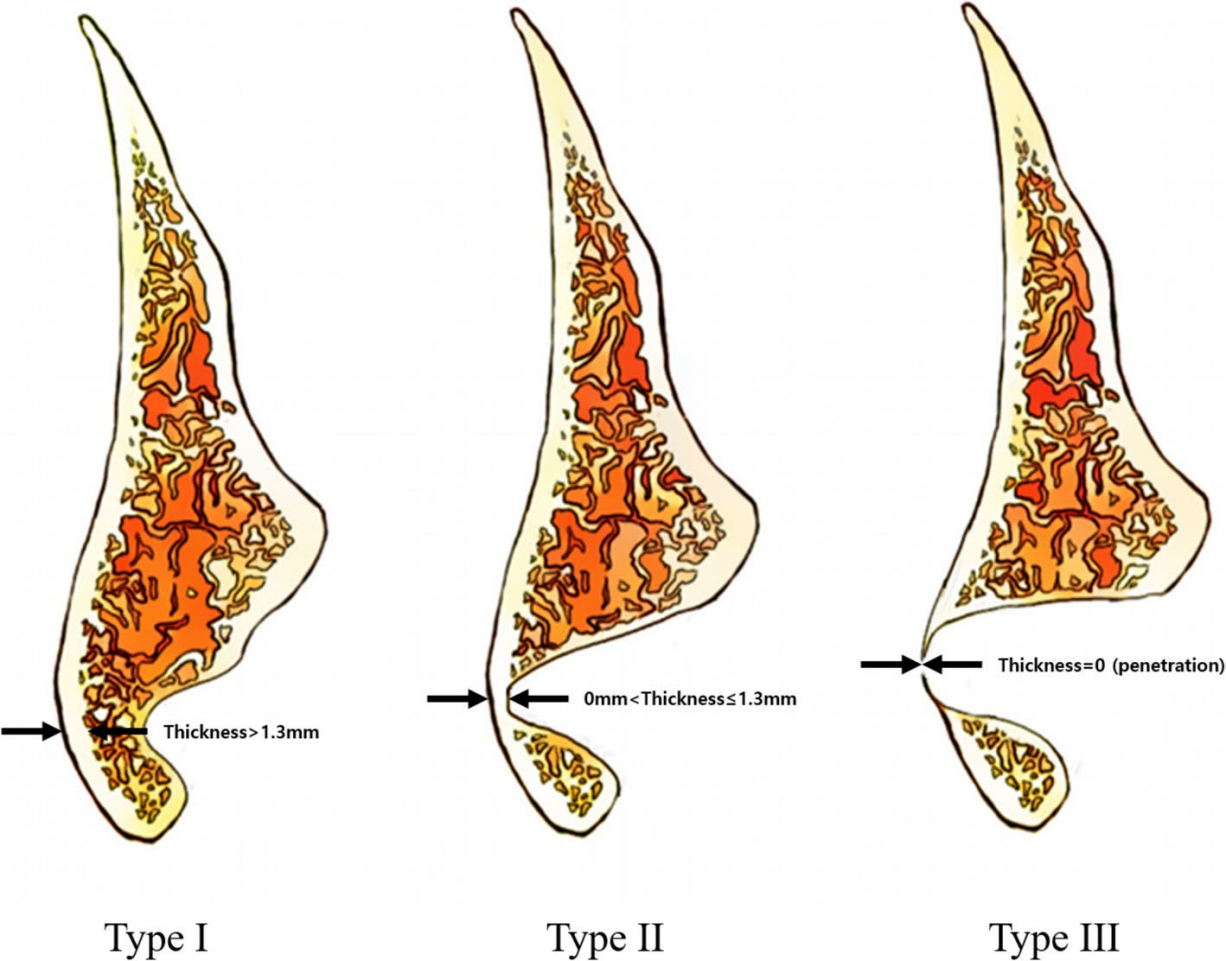
The new classification based on the thickness of buccal cortical bone with a reference thickness of 1.3 mm. In type I, the thickness of buccal cortical bone exceeds 1.3 mm. In type II, the thickness of buccal cortical bone is above 0 mm but not exceeding 1.3 mm. In type III, the thickness of buccal cortical bone is 0 mm

Prior studies have attempted to categorize buccal cortical bone patterns of SMBC using CBCT. Ariji et al. [15] categorized SMBC based on the depth of mandibular bone infiltration measured with computed tomography. However, this classification method did not account for cases where the buccal cortical bone on the affected side was perforated, and it varies depending on the interpretation by the dentist reading the images. Additionally, there are limitations in this approach for periodic follow-up of SMBC, as it cannot provide objective numerical measurements of changes in lesion size. Hence, to objectively assess changes in SMBC during follow-up, a new classification method utilizing measurements of the remaining buccal cortical bone thickness on CBCT is needed. However, there have been no documented classifications based on the actual cortical bone thickness in the literature. The average buccal cortical bone thickness on the affected side of the mandible has been reported as 1.33 ± 0.38 mm [16]. Based on this, SMBC cases can be categorized based on an average buccal cortical bone thickness of 1.3 mm in the affected mandible (Fig. 7).

Numerous prior studies classify the location of SMBC into two primary variants: the posterior variant, found in the posterior region of the mandible, distal of the lower premolars, and the anterior variant, located in the anterior region of the mandible, mesial of the lower premolars. The majority of SMBC cases fall within the posterior variant category, with the anterior variant being a rare occurrence [17]. In all cases of this study, SMBC appeared as the posterior variant, located in the posterior region of the mandible, including the distal area of the lower premolars and the mandibular angle. The rarity of the anterior variant originating in the anterior region of the mandible poses challenges in terms of differential diagnosis, particularly when the radiographic features of SMBC, observed unilocular, overlap with other anatomical structures like the root of the anterior tooth on X-ray images. This overlap can lead to misdiagnosis as odontogenic benign tumors or periapical cysts that commonly occur in the anterior mandible [18, 19]. Regarding the relationship between the mandibular canal and the location of SMBC, this study observed where SMBC includes the mandibular canal in 3 cases (9.38%). This finding is consistent with previous research, which reported a similar result that the majority of SMBC cases are situated below the mandibular canal, with approximately 14.6% of cases showing a pattern that includes the mandibular canal [13].

In typical cases, distinguishing SMBC from other lesions can be achieved by recognizing its clinical characteristics and features on panoramic radiographs and CBCT scans, as described earlier. However, when it remains difficult to differentiate the lesion even with the information provided, consideration should be given to alternative diagnostic methods. One such method is sialography. SMBC with ductal involvement can be visualized in sialography, revealing the ductal structure of the lesion on the imaging [20]. Several studies have reported the observation of portions of salivary gland ducts inside or around SMBC using sialography [20, 21]. This diagnostic approach enables the differentiation of SMBC from other lesions. Another diagnostic method for differential diagnosis is magnetic resonance imaging (MRI). MRI offers excellent soft tissue resolution and contrast, allowing for the observation of internal structures, and vascular patterns, and aiding in the differentiation of SMBC from true cysts containing only fluid. However, it should be noted that MRI can be costly and may impose a financial burden on patients. Additionally, field distortion artifacts can occur due to intraoral dental prostheses, which is a limitation of this imaging modality [22].

Since SMBC is typically asymptomatic and lacks complications, surgical intervention is not necessary. Instead, it requires periodic radiographic follow-up. Lesions suspected to be SMBC should undergo regular radiographic follow-up at intervals of 12 months to monitor any changes in the size and morphology of the lesion [23]. If changes in the lesion are observed during follow-up, it is recommended to consider surgical intervention and tissue biopsy for the purpose of differential diagnosis from other lesions. In one case in this study, surgical intervention and tissue biopsy were performed due to changes in the size of the lesion, aiming to differentiate it from salivary gland tumors. The biopsy revealed lymphocytic sialadenitis. Another indication for surgical intervention is when the defect leads to the weakening of the mandibular bone structure itself. In such cases, to prevent the risk of fractures, it is necessary to remove the soft tissue within the defect and reinforce the mandibular bone structure using materials like titanium plates.

## Conclusion

SMBC is a benign lesion primarily found in the mandibular angle and posterior body of the mandible. In most cases, treatment is not necessary, and differentiation from other lesions can be achieved by understanding its clinical characteristics and features on panoramic radiographs and CBCT. If differentiation becomes challenging based on the provided information, sialography, and MRI imaging can be helpful. However, in cases where changes in the size and morphology of the lesion are observed during follow-up, surgical intervention and tissue biopsy should be considered for differential diagnosis from other lesions. If surgical intervention has been performed, and the lesion within the defect has been removed, reinforce the mandible using grafting materials or titanium to prevent fractures.

## Data Availability

Not applicable.

## Abbreviations

SMBC: Stafne mandibular bone cavity
CBCT: Cone beam computed tomography
MRI: Magnetic resonance imaging
